# Impacts of Climate Change on the Geographic Distribution of *Dioscorea zingiberensis*, a Traditional Medicinal Plant in China

**DOI:** 10.3390/plants15101444

**Published:** 2026-05-09

**Authors:** Ting-Ting Zhang, Xin Jiang, Hao-Ran Yang, Yun Jia

**Affiliations:** 1 College of Life Science and Technology, Gansu Agricultural University, Lanzhou 730070, China; yanghaoran1224@163.com; 2 College of Life Sciences, Northwest University, Xi’an 710069, China; 202133347@stumail.nwu.edu.cn; 3 Xi’an Botanical Garden of Shaanxi Province (Institute of Botany of Shaanxi Province), Xi’an 710061, China

**Keywords:** environmental factors, MaxEnt model, migratory trajectory, species distribution model

## Abstract

*D. zingiberensis* C. H. Wright is a medicinally significant herbaceous vine endemic to China. Investigating the geographical distribution and migration routes of *D. zingiberensis* is crucial for the rational utilization and conservation of its genetic resources. However, the potential shifts in the distribution patterns of wild populations under different climate scenarios remain poorly understood. Based on the MaxEnt model and ArcGIS, this study reveals significant range shifts in *D. zingiberensis* under future climate scenarios. Under SSP1-2.6, highly suitable habitats are projected to occur in Shaanxi, Hubei, Sichuan, and Gansu by the 2050s, with total suitable areas peaking at 211.41 × 10^4^ km^2^. In contrast, the high-emission SSP5-8.5 scenario drives marked habitat contraction, with a core loss of 82.47 × 10^4^ km^2^ by the 2070s, particularly in the central and southwestern provinces (e.g., Chongqing, Sichuan, Hubei, and Hunan). Centroid migration analysis indicates a pronounced northward shift; under SSP5-8.5, the centroid moves linearly northwestward by 205.43 km from Hubei to Sichuan, reflecting a sustained migration towards higher latitudes. These results underscore *D. zingiberensis*’s vulnerability to high-emission climates and its adaptive migration towards more suitable northwestern habitats. These findings provide critical information and a scientific basis for the conservation and sustainable utilization of wild medicinal resources of *D. zingiberensis*.

## 1. Introduction

Global climate change is considered one of the most profound and urgent challenges confronting contemporary society. Observational data indicate that during the decade from 2014 to 2023, the global temperature rose by 0.26 °C, and the average surface temperature was 1.19 °C higher than the pre-industrial baseline of 1850–1900 [1,2,3]. As a fundamental environmental factor governing species distribution and geographic ranges, this rapid climatic change is profoundly reshaping the patterns of global biodiversity. Human activities also constitute a crucial driver of species’ geographic distribution. Concurrently, growing emphasis on plant adaptation to climate change has elevated research on species spatial patterns to a forefront topic in ecological studies [4]. Meanwhile, under global climate change, plant species are expected to undergo geographical distribution shifts in response to climatic oscillations, facing dual pressures from habitat displacement and heightened extinction risks [5,6,7]. Therefore, it is necessary to focus on plant responses to varying climatic scenarios. Studying species-specific responses to climatic shifts under different scenarios would not only help decipher the historical processes underpinning speciation and biogeographic patterning but also inform the formulation of scientific ecosystem management and conservation strategies.

It is well-established that simulating and projecting species’ geographic distributions under climate change scenarios offers a viable framework for elucidating variations in species’ adaptive capacity and forecasting their potential range shifts [8,9]. This framework is predominantly operationalized through species distribution models (SDMs), also termed habitat models, which constitute a cornerstone methodology for assessing species distributions from distribution points and environmental covariates, estimating spatial turnover in species richness, identifying drivers of distributional shifts, and inferring potential migration corridors, thereby directly supporting the quantification of adaptive responses and range dynamics [10].

Advances in computational technology and geographic information systems (GIS) have spurred a proliferation of SDM software tools such as GAPR, CLIMEX, BIOCLIM, and MAXENT [11]. These models exhibit strengths/limitations from divergent theories, with performance unstable under data changes. Among them, MaxEnt, grounded in maximum entropy theory, uses presence-only data, showing robust predictions with limited samples [12,13,14]. Currently, the MaxEnt model has been extensively utilized across diverse disciplines, encompassing conservation biology and invasion biology. For instance, researchers incorporated habitat characteristics and saponin profiles of *Panax notoginseng* to evaluate its potential geographic range using the MaxEnt model [15]. The impacts of climate change on the spatial distribution of the endangered *Rhododendron* species in the Qinba Mountains were revealed by employing the MaxEnt model and phylogeographic methods [16]. Additionally, the model has been integrated with phylogenetic analysis and plastid genomics to evaluate species delimitation and the invasion potential of invasive populations, exemplified by the *Opuntia humifusa* complex in China [17].

*Dioscorea zingiberensis* C. H. Wright, a medicinally significant herbaceous vine endemic to China, has been employed in traditional folk medicine for over two millennia due to its substantial content of diosgenin and diosgenin-type saponins, which can constitute up to 16.15% of its dry weight. It is also globally recognized as a primary botanical source of steroidal hormone precursors [18,19,20]. Generally, *D. zingiberensis* predominantly inhabits valleys and low- to mid-elevation hills at altitudes ranging from 100 to 1500 m. Its typical habitats include deciduous broad-leaved and evergreen broad-leaved mixed forests or forest margins within shrublands [21]. The rhizome of *D. zingiberensis* possesses detoxifying and anti-inflammatory properties, making it effective in the treatment of non-ulcerated furuncles, coronary heart disease, acute suppurative skin infections, and soft tissue injuries [22,23]. The escalating demand for wild *D. zingiberensis* in the medicinal market has led to predatory and indiscriminate harvesting, severely damaging its wild populations and degrading their native habitats [24]. In this context, utilizing ecological niche models to project potential shifts in the geographic distribution and identify migration pathways of *D. zingiberensis* under varying climate scenarios provides a critical tool for elucidating the niche dynamics of its wild populations, offering essential insights for the conservation and sustainable utilization of its wild genetic resources.

Despite the recognized medicinal importance of *D. zingiberensis*, critical knowledge gaps persist regarding the spatiotemporal responses of its wild populations to future climate change. Previous studies have primarily focused on its current distribution or pharmacological properties, lacking comprehensive projections under contrasting emission scenarios. Specifically, quantitative assessments of future habitat shifts, identification of potential migration corridors, and delineation of climate refugia for conservation planning are still insufficient. To address these gaps, this study employs the MaxEnt model to investigate the distribution shifts under current and future scenarios (SSP1-2.6 and SSP5-8.5 for the 2050s, 2070s, and 2090s). Our objectives are to: (1) quantify the key environmental drivers governing its distribution; (2) project potential spatial patterns and habitat suitability across multiple time horizons; (3) evaluate the dynamics of habitat expansion and contraction; and (4) innovatively infer population migration trajectories to identify priority conservation zones. By doing so, this study provides novel insights into the species’ range dynamics under climate change, offering a scientific basis for the targeted conservation and sustainable utilization of its wild medicinal resources.

## 2. Results

### 2.1. Accuracy Evaluation of the Maxent Model

**Figure 1:**
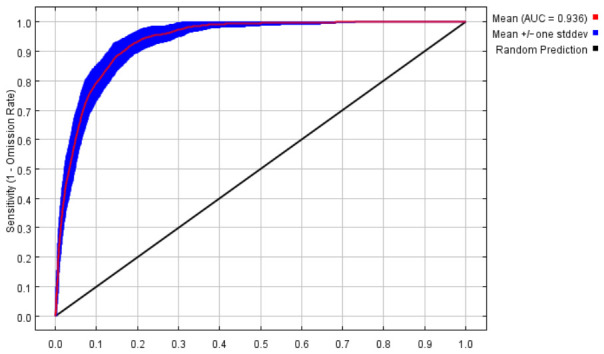
The evaluation of the MaxEnt model accuracy, based on the receiver operating characteristic (ROC) curve, was derived from the average result of the cross-validation procedure conducted on the training dataset.

The MaxEnt model demonstrated high efficacy in forecasting the potentially suitable habitats for *D. zingiberensis*, with the area under the receiver operating characteristic curve (AUC) values illustrated in Figure 1. The average AUC, derived from ten replicate runs, reached 0.936, indicating an excellent predictive performance of the model for the species’ geographic distribution. In addition, the results from Figure S1 (training/test ROC) showed high consistency (AUC: 0.946/0.953), confirming robust generalization of the MaxEnt model. Furthermore, both the TSS and Kappa values alongside the AUC were generated, with a TSS value of 0.772 ± 0.009, indicating robust predictive ability. The low Kappa value (0.129 ± 0.021) was attributed to class imbalance inherent in the presence-background data. Therefore, considering the high AUC value (>0.9) and the “very good” TSS score collectively, we held that the constructed MaxEnt model exhibits good performance and provides reliable predictions.

### 2.2. Analysis of Key Environmental Variables

**Figure 2:**
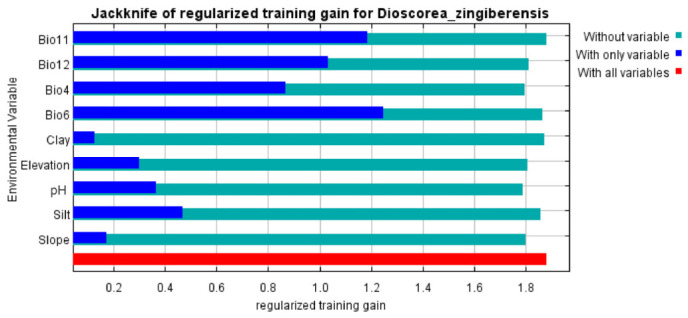
Jackknife test of importance environmental variables.

The variable contribution analysis from the MaxEnt model, complemented by permutation importance and jackknife testing, identified a suite of key environmental factors governing the potential distribution of *D. zingiberensis*. In terms of percent contribution (Table S1), the minimum temperature of the coldest month (Bio6, 36.4%) and the mean temperature of the coldest quarter (Bio12, 35.9%) emerged as the most influential variables, collectively accounting for nearly 72.3% of the total contribution. This was followed by annual precipitation (Bio4, 9.1%), slope (6.0%), elevation (4.6%), soil water pH (pH, 3.0%), silt content (1.9%), clay content (1.7%), and the mean temperature of the wettest quarter (Bio11, 1.4%). Notably, the permutation importance (a metric reflecting a variable’s independent contribution to model predictive power) revealed distinct patterns: temperature seasonality (Bio4, 57.8%) and elevation (24.9%) exhibited substantially higher values, underscoring their critical role in enhancing the model’s discriminatory ability. In contrast, bioclimatic variables (e.g., Bio6: 4.5%, Bio12: 3.9%, Bio11: 1.3%) and topographic/edaphic factors (≤4.2%) demonstrated relatively lower permutation importance. The jackknife test of regularized training gain (Figure 2) further validated the importance of these variables. For example, Bio11 showed a marked increase in training gain when included (blue bar) relative to when it was absent (green bar), indicating its unique contribution to model performance. Similarly, Bio12, Bio4, and Bio6 exhibited considerable gains when individually evaluated, consistent with their high percent contributions. Collectively, climatic variables (particularly Bio6, Bio12, Bio4, and Bio11) dominated the determination of suitable habitat for *D. zingiberensis*, with their importance corroborated by both percent contribution and jackknife-derived training gains (Figure 2 and Figure S2). Topographic (slope, elevation) and edaphic (pH, silt, clay) factors, although less influential in terms of percent contribution, still contributed to predictive accuracy, as evidenced by their non-negligible permutation importance and jackknife performance.

### 2.3. The Contemporary Suitable Habitats of D. zingiberensis

**Figure 3:**
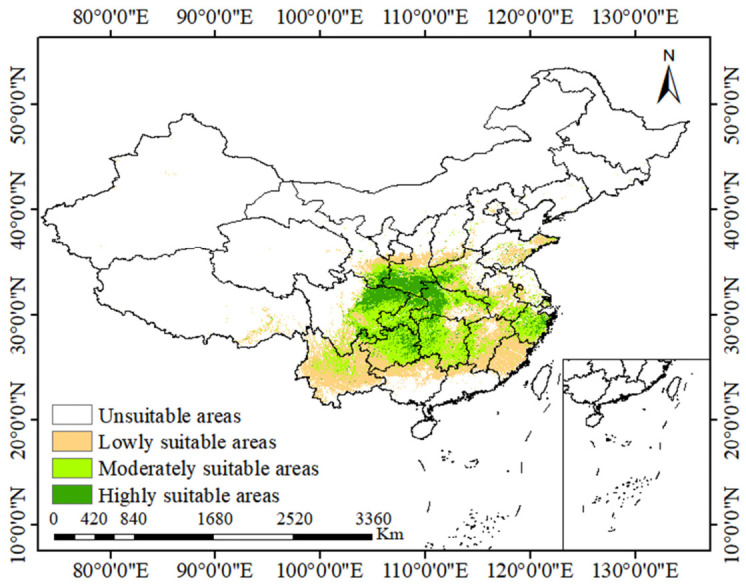
Potentially suitable areas of *Dioscorea zingiberensis* under current climate scenarios.

The MaxEnt model simulation for the current period delineated the potential distribution of *D. zingiberensis* (Figure 3). The predicted suitable habitats were widely distributed across ten provincial-level administrative regions in China, spanning central, southwestern, and northwestern areas, including Hubei, Shaanxi, Sichuan, Chongqing, Guizhou, Gansu, Hunan, Zhejiang, Yunnan, and Fujian (Figure 3). Within this broad range, the core areas of high habitat suitability, covering a total area of approximately 36.28 × 10^4^ km^2^ (Table 1), were concentrated in Shaanxi, Sichuan, Chongqing, and Hubei. This predicted core zone showed strong concordance with the majority of the species’ known occurrence records, validating the model’s output. Furthermore, zones of moderate suitability extended from this core into adjacent regions, notably encompassing parts of Guizhou, Gansu, Hunan, and Zhejiang.

### 2.4. Distribution Patterns in Future Climate Scenarios

**Figure 4:**
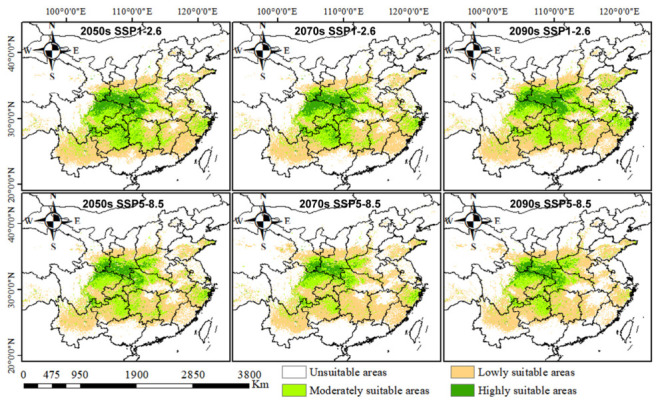
Prediction of potential suitable distribution of *Dioscorea zingiberensis* under future climate scenarios.

To project the future potential distribution of *D. zingiberensis*, we employed MaxEntv 3.4.1 to simulate habitat suitability under two contrasting shared socioeconomic pathway (SSP) scenarios, i.e., SSP1-2.6 and SSP5-8.5, for the 2050s, 2070s, and 2090s (Figure 4). The simulation results revealed distinct spatiotemporal patterns of habitat change under the two scenarios. Under the SSP1-2.6 scenario, regions of high habitat suitability were predominantly projected in Shaanxi, Hubei, Sichuan, and Gansu provinces. The 2050s period exhibited the maximum extent of both highly suitable habitat (27.93 × 10^4^ km^2^) and total suitable area (211.41 × 10^4^ km^2^) (Table 1).

In contrast, under the high-emission SSP5-8.5 scenario, high-suitability areas in the 2050s were also initially concentrated in Shaanxi, Hubei, Gansu, and Sichuan. However, by the 2070s, a notable contraction and spatial consolidation occurred, with high-suitability habitats becoming predominantly confined to Shaanxi, Gansu, and Sichuan, with Shaanxi retaining the largest core area. A significant distributional shift was projected by the 2090s under the SSP5-8.5 scenario, with a portion of the suitable habitat expected to migrate towards Henan and Guizhou provinces (Figure 4). A consistent trend across both scenarios was the projected temporal decline in the total suitable area for *D. zingiberensis*. This overall habitat loss was primarily driven by the progressive contraction of moderate- and high-suitability zones over time (Table 1).

### 2.5. Climate Impacts on the Geographical and Spatial Patterns of D. zingiberensis

**Figure 5:**
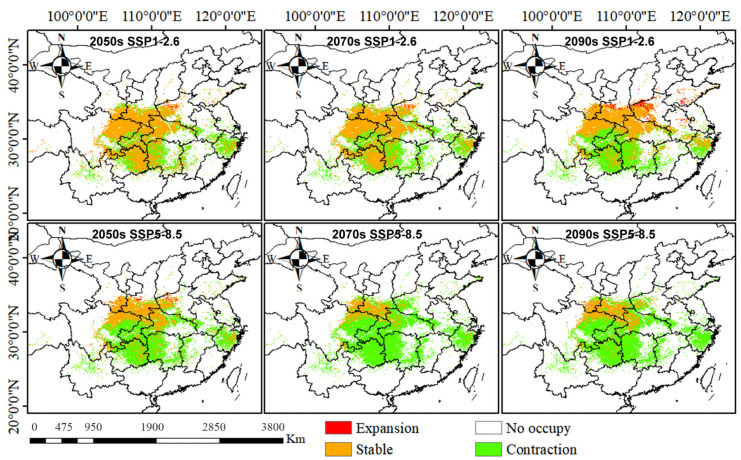
Future spatial and geographical changes under different climate scenarios.

To investigate the effects of climate change on the distribution of *D. zingiberensis* under different scenarios, we simulated the distribution patterns in different periods by comparing future habitat projections with current distributions. The results revealed significant changes in *D. zingiberensis*-suitable habitats. Under the SSP1.26 scenario, habitat expansion was primarily observed in central and western Henan, southern Shaanxi, and southern Shandong (Figure 5). Conversely, under the SSP5.85 scenario, habitat contraction was pronounced, with the most significant reduction occurring in the 2070s, affecting an area of 82.47 × 10^4^ km^2^ (Table 2). The projected habitat contraction primarily affected regions in central and southwestern China, including Chongqing, Sichuan, Hubei, Hunan, Guizhou, and parts of Zhejiang and Jiangxi. Overall, the extent of habitat loss exceeded that of expansion, with notable expansion trends observed in the central-western regions (particularly within the Qinba Mountains) and predominant contraction occurring in the southeastern parts of the study area (Figure 5).

**Figure 6:**
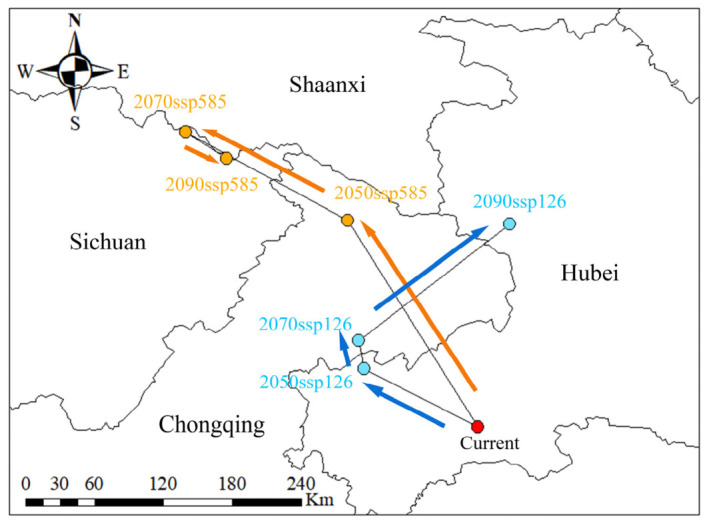
Centroid migration of suitable habitats for *Dioscorea zingiberensis*. Arrows under SSP1-2.6 and SSP5-8.5 scenarios. Arrow symbols indicate migration direction; blue and orange dots mark the distribution centroids for the SSP1-2.6 and SSP5-8.5 scenarios, respectively.

To elucidate the spatial dynamics of suitable habitats under projected future climates, the distribution centroids for each scenario were computed, and their migratory trajectories were delineated (Figure 6). The analysis revealed a pronounced northward shift in the species’ distribution centroid across the different periods. Under the SSP1-2.6 scenario, the centroid exhibited a cyclical migration pattern: it initially shifted from Hubei Province towards Chongqing Municipality between the 2050s and 2070s, subsequently returned from Chongqing to Hubei from the 2070s to the 2090s, and ultimately re-established within Hubei Province by the 2090s. In stark contrast, under the SSP5-8.5 scenario, the centroid demonstrated a more linear and sustained northward displacement, progressing from Hubei to Chongqing in the 2050s, and then further to Sichuan Province by both the 2070s and 2090s (Figure 6).

Under the SSP5-8.5 scenario, the migratory trajectory of the distribution centroid was longer and more complex than that under the SSP1-2.6 scenario. The centroid first shifted northwestward from Hubei Province to Chongqing Municipality, covering a distance of 205.43 km. It then continued moving northwestward at a more pronounced angle for another 142.68 km, reaching Sichuan Province. Finally, after a minor southeastward adjustment of 38.00 km, the centroid stabilized within Sichuan (Table 3, Figure 6).

## 3. Discussion

The MaxEnt algorithm was widely selected for species distribution modeling due to its recognized capacity to produce accurate predictions even with sparse species presence records [25,26,27]. In the present study, the strong agreement between cross-validation and independent test results further demonstrates the model’s robustness and low overfitting risk, supporting its utility for distribution prediction of *D. zingiberensis*. We found that the potential distribution of *D. zingiberensis* is predominantly constrained by a combination of climatic and edaphic-topographic variables. Among the climatic factors, temperature-related parameters—specifically Bio6, Bio4, and Bio11, together with moisture availability as reflected by Bio12—emerged as key determinants. This finding is consistent with other species distribution studies, in which minimum winter temperatures often define critical physiological limits for survival, while precipitation regimes fundamentally constrain growth and regeneration by mediating water stress and seasonal resource availability [28,29,30]. The climatic driver annual precipitation (Bio12) underscored the importance of hydrological conditions in shaping the species’ ecological niche [31]. As the primary determinant of soil moisture availability, Bio12 critically governs plant water balance, shapes phenological rhythms, and ultimately defines the hydrologic niche of species. Its spatial gradients directly limit species’ ranges by imposing thresholds on drought tolerance and regulating the length of favorable growth periods, thereby structuring continental-to-regional scale biodiversity patterns [32].

Apart from temperature and precipitation factors, topographic as well as soil properties exerted a certain degree of influence on the suitable distribution for *D. zingiberensis* (belonging to Dioscoreaceae). These factors govern root architecture, nutrient ion availability, and microbial symbioses. For a rhizomatous medicinal species, soil chemistry may directly influence secondary metabolite production (e.g., steroidal saponins) and be related to the accumulation and transformation of bioactive substances in the species rhizomes, linking habitat suitability to medicinal quality [33,34]. In addition, elevation likely serves as a proxy for integrated climatic gradients, while slope may influence local microclimate, soil drainage, and erosion processes, collectively defining the physiographic context for the species’s suitable habitats [35,36]. This implies that purely climate-driven distribution models might overestimate the suitable area if key soil constraints are not met. Topographic variables (elevation, slope) further refine this niche by integrating microclimatic gradients and drainage conditions, creating the fine-scale heterogeneity that defines actual habitat patches within broader climatically suitable zones [37]. Given that the distribution of *D. zingiberensis* is constrained by a synergistic interplay of temperature, precipitation, topographic, and edaphic factors, playing a particularly defining role in shaping its realized niche, future conservation and cultivation strategies should adopt a multi-dimensional approach focused on preserving and replicating these critical abiotic conditions. Specifically, in situ conservation efforts should prioritize the protection of existing habitats that exhibit optimal soil profiles and favorable topographic features such as mid-elevation slopes with adequate drainage and microclimatic buffering [38]. Furthermore, soil amendment trials and controlled cultivation experiments could help identify tolerable ranges for key soil parameters, informing the restoration of marginal habitats and the sustainable cultivation of *D. zingiberensis* in agroforestry systems.

Projections indicate divergent responses among species to climate change, with some exhibiting potential range expansions and others experiencing contractions. For instance, modeling studies suggest that the climatically suitable habitat range for *Changium smyrnioides* (a perennial herb of significant medicinal value in China) may increase under future warming scenarios [39]. In contrast, the projected high-suitability area for *Paeonia rockii* under the future scenario is estimated to be 16.88% smaller compared to its current extent [40]. Similar to the latter pattern, our MaxEnt model demonstrated competent predictive accuracy, identifying the core suitable habitats for *D. zingiberensis* in the Qinba Mountains and surrounding regions, specifically Shaanxi, Hubei, Sichuan, and Chongqing, aligns with the species’ well-documented status as a plant exclusively cultivated in China with significant medicinal value [41,42,43]. The projected future distribution shifts under the SSP5-8.5 scenario, characterized by a migration towards higher latitudes or specific refugia like Henan and Guizhou, reflect a broader trend observed in plant species distribution modeling. As noted in studies on spruce species, plants often track shifting climatic gradients, moving towards areas with suitable temperature and precipitation regimes [44,45]. The contraction of suitable areas under high-emission scenarios is a critical concern, echoing findings for other vulnerable species where high fossil fuel usage scenarios lead to substantial decreases in habitat suitability [46,47]. This loss of habitat is particularly alarming given the existing genetic structure of *D. zingiberensis*; previous research has shown that the species possesses significant genetic differentiation among populations. Habitat fragmentation caused by climate change could further impede gene flow, which is already limited among certain populations, thereby increasing the risk of genetic erosion [48,49,50]. Therefore, conservation strategies must prioritize the protection of these core refugia, including the Qinba Mountains, by implementing vegetation and soil conservation measures, maintaining habitat connectivity, and avoiding infrastructure development, such as roads that lead to habitat fragmentation, to ensure the persistence of the species’ genetic diversity and its continued availability as a medicinal resource.

The projected northward shift and contraction of suitable habitats for *D. zingiberensis* under high-emission scenarios align with global patterns observed in other medicinal plant species. Analogous to our findings under the SSP5-8.5 scenario, where the distribution centroid migrated towards higher latitudes, studies on Himalayan medicinal plants have documented significant upward and northward distribution shifts as species track suitable climatic conditions [51,52]. This phenomenon reflects a broader ecological response where plants attempt to adapt to warming climates by moving to cooler, high-latitude or high-elevation refugia [53,54,55]. However, the severe habitat contraction observed, particularly the loss of suitability in southeastern regions, corroborates findings for *Zingiber* species in China, which are projected to experience dramatic decreases in suitable habitat area due to climate change [33]. Similar distributional shifts have been documented in other related taxa; for instance, studies on *Alpinia officinarum* revealed a comparable trend wherein suitable habitats significantly contracted and shifted northwestward under high-emission scenarios, with a pronounced reduction of approximately a quarter observed specifically under the SSP5-8.5 scenario [56].

The contrasting trajectories between SSP1-2.6 and SSP5-8.5 scenarios underscore the critical impact of emission pathways on species persistence; the sustained displacement under SSP5-8.5 suggests that *D. zingiberensis* may face increasing physiological stress and habitat fragmentation, potentially leading to a reduction in resource availability similar to the decline noted in wild *Dioscorea* populations due to environmental pressures [57]. To address the projected habitat contraction and northward shift of *D. zingiberensis*, conservation strategies should integrate robust in situ and ex situ measures. Specifically, this involves identifying and reinforcing in situ refugia, such as the Qinba Mountains, which can buffer against climatic extremes by leveraging topographic microclimates and maintaining habitat connectivity to ensure population persistence. Concurrently, developing environmentally sustainable ex situ cultivation systems is crucial. This includes optimizing propagation techniques and adopting cleaner production methods, such as enzymatic hydrolysis or supercritical fluid extraction, to reduce the ecological footprint of diosgenin processing and establish a reliable supply chain independent of vulnerable wild populations.

Beyond geographic range shifts, climate change may also alter the medicinal quality of *D. zingiberensis*, which is primarily valued for its steroidal saponins like diosgenin. The biosynthesis and accumulation of these secondary metabolites are highly sensitive to environmental stresses, including temperature fluctuations and water availability [58]. Furthermore, projected increases in temperature and alterations in precipitation patterns under high-emission scenarios could induce physiological stress, potentially disrupting the biosynthetic pathways of diosgenin. Studies on other medicinal plants have shown that drought or heat stress can lead to either an increase or a decrease in specific secondary metabolites, often at the cost of growth [59]. Therefore, the northward migration of suitable habitats may not only represent a spatial shift but also relocate populations to environments where the delicate balance for optimal diosgenin production is altered. Future conservation and cultivation efforts must consider this dual challenge: safeguarding populations in stable refugia (like the Qinba Mountains) where both survival and traditional medicinal quality might be preserved, and selectively breeding or managing ex situ cultivations in new suitable areas to maintain or enhance the desired phytochemical profile under changed climates.

Nevertheless, several limitations of this study should be considered. First, the projections rely on a single global climate model (BCC-CSM2-MR), introducing uncertainty, as different models can yield varied future climate patterns. Second, potential sampling bias in occurrence records may exist. Third, the MaxEnt model uses presence-only data and assumes equilibrium between the species and its environment, without accounting for biotic interactions or dispersal limitations. Consequently, the future predictions carry inherent uncertainty and should be interpreted as potential distributional trends rather than definitive forecasts.

## 4. Materials and Methods

### 4.1. Collection of Occurrence Data

**Figure 7:**
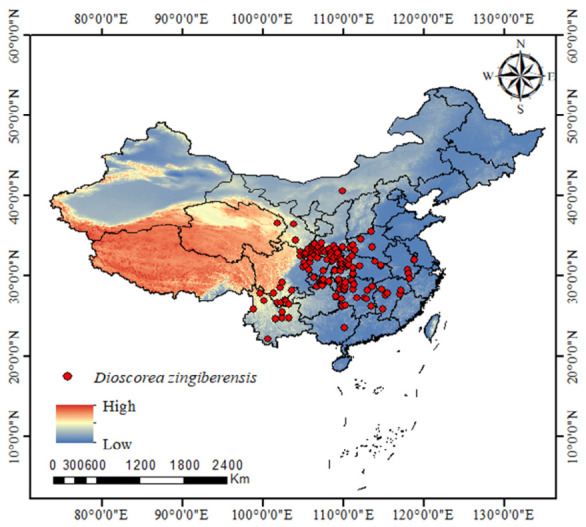
Spatial distribution of *Dioscorea zingiberensis*. The geographical distribution of *Dioscorea zingiberensis* is indicated by red dots. Please note that ‘High–Low stretch’ refers to the elevation above sea level.

*Dioscorea zingiberensis* C. H. Wright, commonly known as ginger yam, is an exclusively cultivated perennial vine in China. To comprehensively delineate the known geographic distribution of the species, occurrence records were obtained from multiple biodiversity data repositories, including the Global Biodiversity Information Facility (GBIF, https://www.gbif.org, Available online: https://doi.org/10.15468/dL.6suhyw, accessed on 17 October 2025), from which only photograph-supported records of *D. zingiberensis* were selected to minimize misidentification (42 distribution points were obtained), the Chinese Virtual Herbarium (CVH, http://www.cvh.ac.cn, accessed on 20 November 2025, 15 distribution points were obtained), and the National Specimen Information Infrastructure (NSII, http://www.nsii.org.cn/2017/home-en.php, accessed on 22 November 2025, 105 distribution points were obtained). SDM toolbox (version 2.0) was employed to mitigate the impact of sampling bias by removing spatially redundant records within a 5 km^2^ area [60]. Following this spatial filtering, a final set of 147 records was retained for subsequent visualization and spatial analysis using ArcGIS 10.8 (ESRI, Redlands, CA, USA) (Figure 7).

### 4.2. Environmental Variables for Analysis

**Figure 8:**
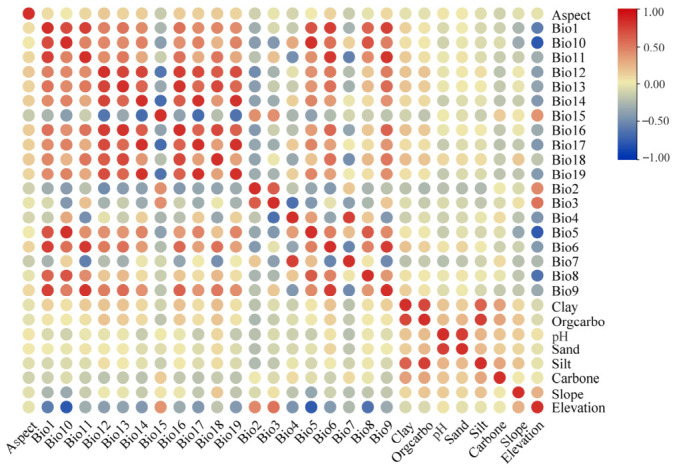
The correlation analysis among various factors. Correlation coefficients between two variables increase as the color intensifies, with red indicating strong correlations and blue denoting weak ones.

The MaxEnt modeling approach was selected for this study due to its proven effectiveness with presence-only occurrence data and its robust performance with relatively small sample sizes [26]. Here, the MaxEnt model was initially constructed using a total of 28 environmental factors (Table 4) at a 2.5 arc-minute (approximately 5 km^2^) spatial resolution using MaxEnt version 3.4.1 [61]. Among these, 19 bioclimatic and 3 topographical variables were obtained from the BCC-CSM2-MR model (considered to be suitable for assessing climate change impacts in China) in the World Climate Database (WorldClim, http://worldclim.org/), encompassing the Last Interglacial, the Last Glacial Maximum, and four additional periods: the Current (1970–2000), the future 2050s (2040–2060), the future 2070s (2060–2080), and the future 2090s (2080–2100). For the future periods, projections under four shared socioeconomic pathways (SSPs), i.e., SSP1-2.6, SSP2-4.5, SSP3-7.0, and SSP5-8.5, were included to represent diverse socioeconomic development trajectories. These pathways are employed to project and understand the impacts of climate change under varying socioeconomic conditions [62]. The MaxEnt model was subsequently applied to predict the potential distribution of *D. zingiberensis* across these different periods and to quantify its projected range expansion and contraction under the various climate change scenarios. Six soil environmental variables (Clay, Orgcarbo, pH, Sand, Silt, and Carbone) were sourced from the Institute of Geographic Sciences and Natural Resources Research, Chinese Academy of Sciences (http://www.resdc.cn) in constructing the MaxEnt model. To mitigate potential overfitting and enhance model interpretability, Spearman correlation analysis (chosen for its robustness to non-normal data distributions and ability to detect monotonic nonlinear relationships) was implemented prior to final model calibration (Figure 8). The Spearman correlation analysis was performed using IBM SPSS Statistics software (Version 26.0). The visualization of correlations among environmental variables was generated using the ggplot2 package in R version 4.3.2 [63]. Specifically, variable pairs exhibiting a Spearman’s rank correlation coefficient absolute value greater than |0.7| were identified. From each such pair, the variable demonstrating the lower percent contribution was subsequently excluded [64]. Following this screening, the nine variables indicated in bold in Table 4 were retained for the definitive model construction.

All environmental factors were treated as continuous variables. The percentage contribution, permutation importation, and jackknife test were employed to assess the relative importance and the unique information contributed by each variable [65]. Response curves were generated to elucidate the relationship between species occurrence probability and each environmental predictor, thereby identifying optimal suitability ranges. For model training and evaluation, 25% of the occurrence records were randomly set aside as an independent test dataset. To ensure robustness and stability of the predictions, the model was run with 1000 iterations, and this procedure was repeated 10 times [61]. Other model parameters were under default settings. The continuous suitability output was converted into a binary map using the ‘10 percentile training presence’ threshold rule, which excludes 10% of occurrence records with the lowest predicted values to mitigate the influence of potentially atypical habitats.

### 4.3. Model Establishment and Performance Evaluation

The receiver operating characteristic (ROC) curve was utilized to assess model performance through calculation of the area under the ROC curve (AUC), as the AUC exhibits reduced sensitivity to sample size and classification threshold variations [60]. The AUC value, which theoretically ranges from 0 to 1, provides a scalar measure of the model’s ability to discriminate between classes, with higher values signifying superior predictive accuracy. A common framework for interpreting AUC values was applied: an AUC exceeding 0.9 was considered indicative of excellent model performance; values between 0.8 and 0.9 denoted good performance; scores from 0.7 to 0.8 suggested moderate performance; and values below 0.7 were regarded as poor [66]. The MaxEnt model was configured with the following settings to ensure robustness and ecological interpretability: for the number of background points, 10,000 points were randomly generated across the study area to comprehensively characterize the available environmental space; for feature classes, a combination of linear (L), quadratic (Q), and hinge (H) features was used, while product (P) and threshold (T) features were excluded to prevent overfitting and maintain model parsimony; for regularization multiplier, this was set to the default value of 1 to appropriately penalize model complexity and promote generalization. To elucidate the drivers of the model’s predictions, a jackknife test was conducted to assess the relative importance of environmental variables in shaping the potential distribution of *D. zingiberensis*. Furthermore, response curve analysis was utilized to quantify and visualize the individual impact of each key environmental factor on the predicted habitat suitability.

### 4.4. Classification of Suitable Habitat

For the investigation of *D. zingiberensis* habitat suitability across multiple temporal scenarios, the continuous probability surfaces generated by the MaxEnt model were utilized as the primary predictive outputs in ASCII format, and then they were imported into ArcGIS 10.8 and converted into raster datasets to facilitate spatial analysis [60]. Subsequently, to delineate distinct habitat suitability classes, the continuous probability values (ranging from 0 to 1, representing the likelihood of species presence) were categorized using the reclassify tool within the same software environment. The resulting potential distribution was stratified into four tiers: areas with a probability (p) greater than 0.7 were classified as highly suitable habitat, *p*-values between 0.5 and 0.7 indicated a moderately suitable habitat, values from 0.3 to 0.5 denoted low suitability, and *p*-values below 0.3 were deemed as an unsuitable habitat [67].

### 4.5. Analysis of Distribution Change

Based on the spatial analysis module in ArcGIS, areas with varying degrees of habitat suitability were delineated. The areal extent of these suitability classes across different time periods was subsequently quantified and compared with the contemporary distribution. Furthermore, the SDM toolbox was utilized to compute the geographic centroids of suitable habitats for each temporal scenario [67]. Finally, potential migratory routes for the population were modeled and visualized within ArcGIS to elucidate the spatiotemporal dynamics of *D. zingiberensis* distribution from historical baselines through to future projections.

## 5. Conclusions

This study employed the MaxEnt model to simulate the distribution of *Dioscorea zingiberensis* under current and future climate scenarios. The results indicate that the minimum temperature of the coldest month (Bio6), annual precipitation (Bio12), temperature seasonality (Bio4), and the mean temperature of the coldest quarter (Bio11) are the most influential climatic factors determining the species’ distribution. Additional physicochemical factors, including slope, elevation, silt content, and soil water pH, also play a defining role in shaping its realized niche, likely influencing both habitat suitability and medicinal quality. The results revealed that the core suitable habitats of *D. zingiberensis* are concentrated in the Qinba Mountains and surrounding provinces (especially Shaanxi, Hubei, Sichuan, and Chongqing), aligning with its known medicinal cultivation range. Future projections under high-emission scenarios (e.g., SSP5-8.5) indicate a concerning northward shift and contraction of suitable habitats. This habitat contraction and fragmentation pose a significant threat to the species’ genetic diversity and resource availability. Therefore, adaptive conservation measures should be implemented, prioritizing the in situ protection of core refugia within the Qinba Mountains to preserve genetic diversity, while promoting ex situ cultivation in areas meeting the species’ specific soil and climatic thresholds to ensure sustainable medicinal utilization. Overall, these findings provide essential insights and a scientific basis for developing effective strategies to ensure the sustainable use and conservation of wild *D. zingiberensis* medicinal resources.

## Figures and Tables

**Table 1:** Suitable habitat areas of *Dioscorea zingiberensis* under different climate change projections (×10^4^ km^2^).

Scenarios	Unsuitable Areas	Lowly Suitable Areas	Moderately Suitable Areas	Highly Suitable Areas	Total Suitable Areas
Current	752.64	96.65	74.43	36.28	207.36
2050s SSP1-2.6	748.59	112.43	71.05	27.93	211.41
2070s SSP1-2.6	760.36	109.53	63.08	27.03	199.64
2090s SSP1-2.6	752.63	114.99	65.99	26.39	207.37
2050s SSP5-8.5	768.86	115.39	56.31	19.45	191.15
2070s SSP5-8.5	769.88	132.16	44.03	13.93	190.12
2090s SSP5-8.5	779.89	118.99	45.11	15.99	180.09

**Table 2:** Potential changes in the distribution areas of *Dioscorea zingiberensis* under different scenarios.

Period	Expansion (×10^4^ km^2^)	Stable (×10^4^ km^2^)	Contraction (×10^4^ km^2^)
Current vs. 2050s SSP1-2.6	1.16	67.45	32.19
Current vs. 2070s SSP1-2.6	0.31	59.65	39.99
Current vs. 2090s SSP1-2.6	3.29	54.69	44.95
Current vs. 2050s SSP5-8.5	0.73	39.80	59.84
Current vs. 2070s SSP5-8.5	0.05	17.17	82.47
Current vs. 2090s SSP5-8.5	0.14	23.66	75.98

**Table 3:** Geometric center of *Dioscorea zingiberensis* and its migration distance under different climate scenarios.

Period	Longitude (°)	Latitude (°)	Migration Direction	Distance (km)
Current	109.899	30.0506	Current→2050ssp126	99.76
2050s SSP1-2.6	109.005	30.5077	2050ssp126→2070ssp126	24.86
2070s SSP1-2.6	108.96	30.7279	2070ssp126→2090ssp126	152.20
2090s SSP1-2.6	110.149	31.6438	Current→2050ssp585	205.43
2050s SSP5-8.5	108.875	31.6756	2050ssp585→2070ssp585	142.68
2070s SSP5-8.5	107.599	32.3656	2070ssp585→2090ssp585	38.00
2090s SSP5-8.5	107.924	32.1624	/	/

**Table 4:** Environmental variables used in this study. The variables selected for constructing the model and predicting the potentially suitable distribution of *Dioscorea zingiberensis* are in bold.

Category	Variable	Description	Unit
Climate	Biol	Annual Mean Temperature	°C
	Bio2	Mean Diurnal Range (mean of monthly (max temp-min temp))	°C
	Bio3	Isothermality (bio2/bio7) (×100)	/
	**Bio4**	Temperature Seasonality (standard deviation × 100)	/
	Bio5	Max Temperature of the Warmest Month	°C
	**Bio6**	Min Temperature of the Coldest Month	°C
	Bio7	Temperature Annual Range (bio5-bio6)	°C
	Bio8	Mean Temperature of the Wettest Quarter	°C
	Bio9	Mean Temperature of the Driest Quarter	°C
	Bio10	Mean Temperature of the Warmest Quarter	°C
	**Bio11**	Mean Temperature of the Coldest Quarter	°C
	**Bio12**	Annual Precipitation	mm
	Bio13	Precipitation of the Wettest Month	mm
	Bio14	Precipitation of the Driest Month	mm
	Bio15	Precipitation Seasonality (coefficient of variation)	/
	Bio16	Precipitation of the Wettest Quarter	mm
	Bio17	Precipitation of the Driest Quarter	mm
	Bio18	Precipitation of the Warmest Quarter	mm
	Bio19	Precipitation of the Coldest Quarter	mm
Topography	**Elevation**	Elevation	m
	**Slope**	Slope	°
	Aspect	Aspect	rad
Soil	**Clay**	/	%
	Sand	/	%
	**Silt**	/	%
	Orgcarbo	Soil Organic Carbon	%
	Carbone	/	%
	**pH**	Hydrogen ion concentration (acidity/alkalinity)	pH

## Data Availability

Data available on request from the authors.

## Supplementary Materials

The following supporting information can be downloaded at: https://www.mdpi.com/article/10.3390/plants15101444/s1, Table S1: Percent contribution and permutation importance of variables; Figure S1: ROC curves derived from the training and independent test datasets; Figure S2: Assessment of environmental variable importance using the jackknife test. (A) test gain, and (B) the area under the receiver operating characteristic curve (AUC).

## Abbreviations

Bio6, Minimum Temperature of the Coldest Month; Bio11, Mean Temperature of the Coldest Quarter; Bio12, Annual precipitation; Bio4, Temperature Seasonality; SSP126, shared socioeconomic pathway 126—this scenario with 2.6 W/m^2^ by the year 2100 is a remake of the optimistic scenario RCP2.6 and was designed with the aim of simulating a development that is compatible with the 2 °C target. SSP585, shared socioeconomic pathway 585—with an additional radiative forcing of 8.5 W/m^2^ by the year 2100, this scenario represents the upper boundary of the range of scenarios described in the literature.
