# Supplementary figures and images for: Impacts of Climate Change on the Geographic Distribution of Dioscorea zingiberensis, a Traditional Medicinal Plant in China
Source: Plants (Basel). 2026 May 9;15(10):1444. doi: 10.3390/plants15101444 (PMC13210834; doi:10.3390/plants15101444)

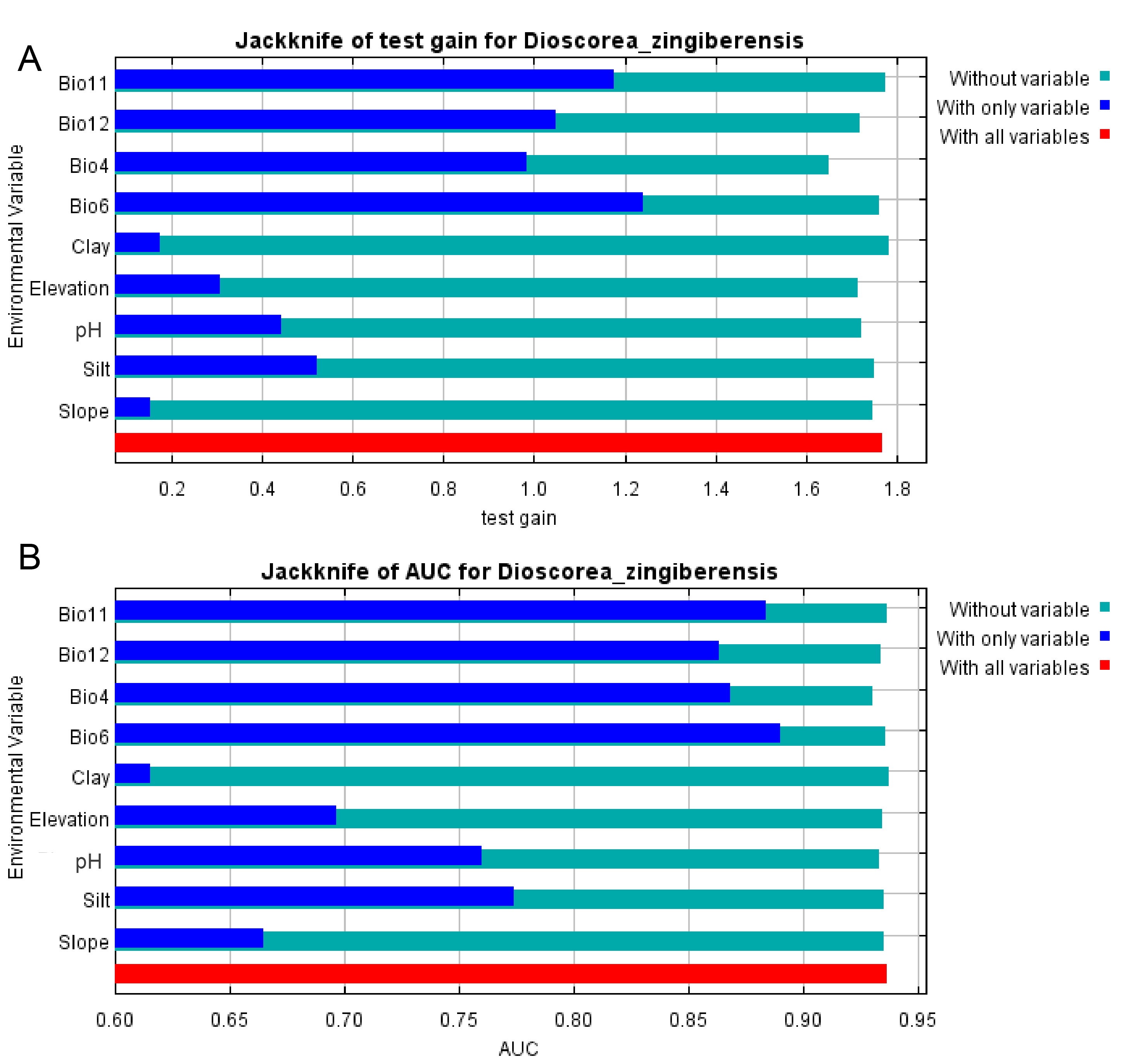

Supplement: Supplementary file 1 [file plants-15-01444-s001.zip › Figure S2.png]

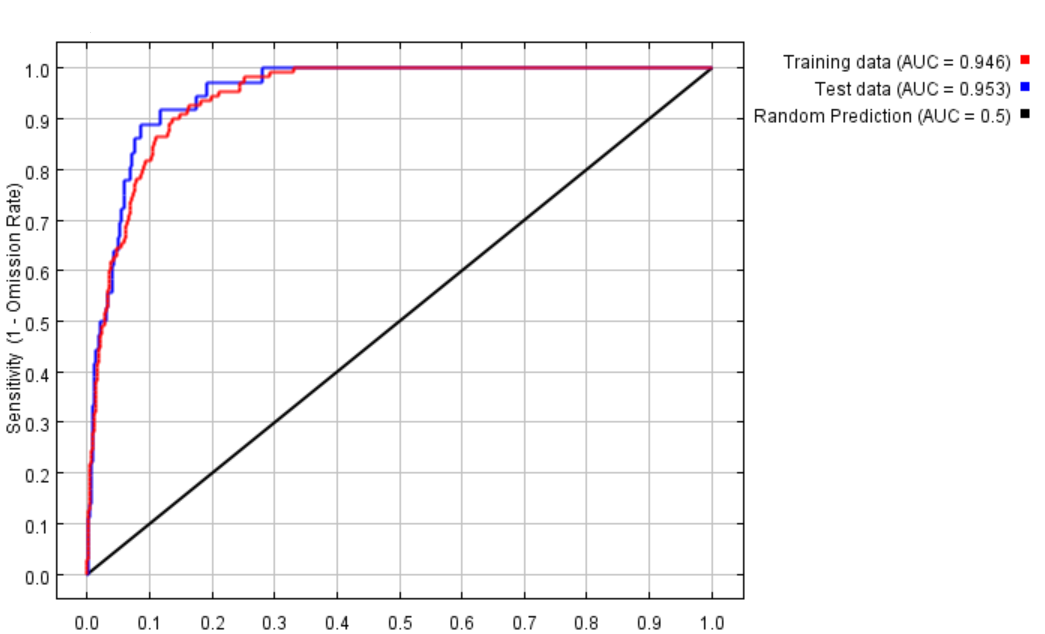

Supplement: Supplementary file 1 [file plants-15-01444-s001.zip › Fiigure. S1.png]
